# Carcinome basocellulaire: à propos d’un cas historique

**DOI:** 10.11604/pamj.2018.30.33.14379

**Published:** 2018-05-16

**Authors:** Mohamed El Amraoui, Mohammed Boui

**Affiliations:** 1 Service de Dermatologie-Vénéréologie, Hôpital Militaire d'Instruction Mohammed V, Rabat, Maroc

**Keywords:** Carcinome baso-cellulaire, infiltrant, naso-orbitaire, Basal cell carcinoma, infiltrating, naso-orbital

## Abstract

We here report the case of an 80 year old man, with clear phototype, villager, residing in one of the most isolated places in our kingdom, located at an altitude above 2400 metres. During humanitarian aid, he presented with ulcero-necrotic tumor infiltrating the nasal root and the right orbit with total destruction of the right eye. The patient reported that the lesion had progressed for two and a half years from a small bleeding papule on the nasal root with orbital spread. Clinical and histological appearance suggested the diagnosis of infiltrating basal cell carcinoma. Staging showed right naso-orbital bone invasion. Mutilating cancer surgery was indicated but the patient refused it. Basal cell carcinoma is reported to have a relatively good prognosis because of its slow and local evolution. However, some cancer types, such as infiltrative tumor occurring in some high-risk areas, including the naso-orbital region, can be rapidly devastating, mutilating and voluminous with aesthetic and functional impairements as well as difficult to manage.

## Image en médecine

Un homme âgé de 80 ans, villageois, résidant dans l'une des zones les plus enclavées de notre royaume, située à plus de 2400 mètres d'altitude, et ayant un phototype clair (II). A consulté, lors d'une mission humanitaire, pour une tumeur ulcéro-nécrotique, infiltrant la racine du nez et l'orbite droits avec destruction totale de l'œil droit. La lésion évoluait, selon le patient, depuis deux ans et demi, par une petite papule saignante sur la racine du nez avec une extension orbitaire. L'aspect clinique et l'histologie étaient en faveur d'un carcinome basocellulaire infiltrant. Le bilan d'extension montrait un envahissement des plans osseux de la région naso-orbitaire droite. Une chirurgie carcinologique et mutilante a été indiquée mais que le patient a refusé. Le carcinome basocellulaire est réputé par son pronostic relativement bon, vue son évolution lente et locale, cependant, certaines formes notamment la forme infiltrante, dans certaines zones à haut risque, notamment dans la zone naso-orbitaire peuvent être rapidement délabrantes, mutilantes et spectaculaires avec des préjudices esthétiques et fonctionnelles et des difficultés de prise en charge.

**Figure 1:**
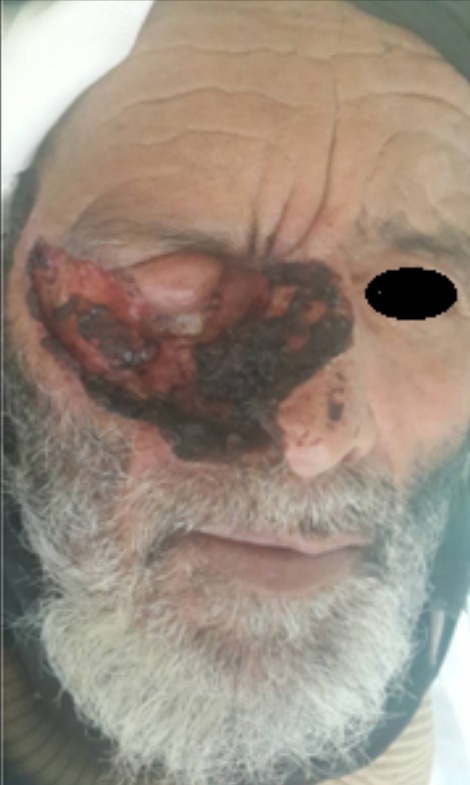
carcinome basocellulaire infiltrant de la région naso-orbitaire droite

